# Corneal Biomechanical and Tomographic Signatures for Keratoconus

**DOI:** 10.3390/diagnostics16213506

**Published:** 2026-10-26

**Authors:** Ariadna Patricia Nicula, Cristina Ariadna Nicula, Dorin Nicula, Adriana Elena Bulboacă, Valeria Coviltir, Sorana D. Bolboacă

**Affiliations:** 1 Medical Informatics and Biostatistics, Department 11—Medical Education, Faculty of Medicine, “Iuliu Hațieganu” University of Medicine and Pharmacy, Louis Pasteur Str., No. 6, 400349 Cluj-Napoca, Romania; nicula.ariadnapatricia@elearn.umfcluj.ro; 2 Oculens Clinic, Calea Turzii, No. 134, 400347 Cluj-Napoca, Romania; cristina.nicula@umfcluj.ro (C.A.N.);; 3 Medical Specialties, Department 1—Maxillo-Facial Surgery and Radiology, Faculty of Dental Medicine, “Iuliu Hațieganu” University of Medicine and Pharmacy, Victor Babeş Str., No. 2–4, 400012 Cluj-Napoca, Romania; 4 Pathophysiology, Department 1—Morpho-Functional Sciences, Faculty of Medicine, “Iuliu Hațieganu” University of Medicine and Pharmacy, Victor Babeş Str., No. 2–4, 400012 Cluj-Napoca, Romania; adriana.bulboaca@umfcluj.ro; 5 Ophthalmology, Department 12—Ophthalmology and Otorhinolaryngology (ENT), Faculty of Medicine, “Carol Davila” University of Medicine and Pharmacy, Boulevard Eroii Sanitari No. 8, Sector 5, 050474 Bucharest, Romania

**Keywords:** keratoconus (KCN), corneal tomography, corneal biomechanics

## Abstract

**Objectives**: This study aimed to identify tomographic and biomechanical biomarkers that differentiate mild/moderate from advanced/severe keratoconus (KCN) based on the ABCD classification. The secondary objective was to assess the diagnostic performance of previously reported biomarkers for distinguishing KCN from KCN-free controls and to compare classifications derived from cohort-specific and previously established cutoff values, evaluating their consistency and potential generalizability in the Romanian KCN population. **Methods**: Adult patients who presented to the Oculens Clinic between 1 January 2024 and 1 September 2025 for clinical care were eligible for enrolment in this observational study. Diagnosis of KCN was made following current practice, and all participants underwent ophthalmologic examination followed by corneal tomography and biomechanical measurements. **Results**: Forty-four subjects with KCN and 38 controls, with a median age of 26.5 years. Patients with mild/moderate KCN (23 patients) were similar in terms of age and sex to those with advanced/severe KCN (21 patients). The values of Kmax showed the best performance in terms of AUC (0.908, 95%CI = [0.824 to 0.992], Se = 76.19 [52.83 to 91.78], Sp = 86.96 [66.41 to 97.22], cutoff = 54.15), with an excellent discriminatory ability in the identification of patients with mild/moderate KCN compared to advanced/severe KCN. Low TL values indicate advanced/severe disease, with the highest values of true positives (18), true negatives (22), and the smallest value of false negatives (3) but with a moderate AUC (0.880 [0.755 to 1.0]). The performances of previously reported cutoff values on the Romanian cohort were tested in 44 patients with KCN and 38 KCN-free individuals. The discriminatory abilities of the previously reported indices, except for KI and BAD-D which were overfitted, were retained in the current cohort, and all exhibited excellent (AUC lower bounds higher than 0.9) or good (AUC lower bounds higher than 0.8) discriminatory performances. The agreement between the current cutoff values and the classification generated by the previously reported cutoff values is sustained by almost perfect agreement in the classification as KCN or KCN-free (Cohen’s kappa values ≥ 0.81, and raw agreement ≥ 90%). **Conclusions**: Our findings support further evaluation of Kmax and corneal thinnest location as markers of keratoconus severity and as potential tools for disease detection. The high concordance observed between classification based on cohort-specific and previously established thresholds suggests consistent performance of these biomarkers within the study population. These parameters may represent promising tools for the management of patients with keratoconus, pending further validation.

## 1. Introduction

Keratoconus (KCN) is a bilateral, non-inflammatory, asymmetric corneal disease characterized by progressive thinning and conical protrusion, resulting in irregular astigmatism, myopia, and reduced visual acuity in its late stages [1]. The onset of the illness occurs in teenagers with rapid progression during the second decade of life [1]. Several pathogenic factors have been implicated in the development of KCN, including genetic factors, eye rubbing, atopy, structural corneal alterations, sex hormones, and the use of hard contact lenses [2]. Research suggests that inflammation may play a key role in the etiology of the disease, as evidenced by the presence of abnormal levels of inflammatory cytokines, such as interleukins (IL-4, IL-5, and IL-8) and tumor necrosis factor (TNF-α) in the tear film and cornea of patients with KCN [3,4,5,6].

Tomographic shape indices and biomechanical (deformation response) indices such as BAD-D, Kmax, CBI (Corvis Biomechanical Index), TBI (Tomography and Biomechanical Index), SP-A1, ARTh, DA ratios, ARC/PRC, TCT and related composites distinguished KCN from normal eyes for cohort-specific cutoff values [7,8,9,10,11,12,13,14,15,16,17,18,19,20].

Tomographic indices used for keratoconus (KCN) assessment quantify changes in anterior and posterior corneal elevation (e.g., Kmax—maximum anterior keratometry) and the pattern of pachymetric thinning. These parameters are frequently reported alongside the Belin/Ambrósio Enhanced Ectasia Display (BAD-D) and the ABCD grading system in both clinical and subclinical KCN populations [9,21,22]. Alternative classification approaches have also been proposed. For example, the Screening Corneal Objective Risk of Ectasia (SCORE) Analyzer software has shown utility in keratoconus detection, but its reported sensitivity and specificity remain moderate [23]. Furthermore, SS-OCT (swept-source optical coherence tomography) derived anterior and posterior elevation measurements, as the thinnest point under the best-fit sphere (BFS) and toric-ellipsoid (BFT), have demonstrated higher specificity than sensitivity for distinguishing keratoconus from normal eyes and keratoconus suspect eyes [24].

Biomechanical indices (deformation response) measure how the cornea reacts to a rapid air-puff test (dynamic corneal response parameters via the Corvis ST device) and tracking metrics such as deformation amplitude and stiffness. The Corvis Biomechanical Index (CBI) is built entirely on these mechanical properties [25]. The Tomographic and Biomechanical Index (TBI) has been reported to provide high discriminative performance for detecting ectatic disease, including subclinical KCN, when integrated with CBI and BAD-D measures [7,8,9,11,21,22,26,27].

While many studies support the superior diagnostic performance of integrated tomography–biomechanics indices (e.g., TBI) over tomographic indices alone, some reports note that certain eyes may show biomechanical abnormalities without overt tomographic changes, underscoring the need for longitudinal follow-up and cautious interpretation in clinical decision-making [6,21,28].

The performance of individual metrics can vary by cohort; for example, CBI and TBI values, cutoffs, and their relative sensitivity/specificity may differ across studies and populations, and some reports emphasize that the “gold standard” remains ensemble metrics rather than a single index [7,8,9,11,14].

Unilateral KCN can show regular topography but abnormal biomechanical indices in a substantial subset, illustrating that biomechanics may reveal subclinical risk not captured by shape alone; prevalence figures for abnormal biomechanics in fellow eyes vary across studies but are repeatedly reported as non-negligible (e.g., up to a notable fraction in some cohorts) [6,21,27,28].

Altered corneal shape and biomechanics measured using Pentacam and Corvis ST have also been reported in the Romanian population, with slightly different cutoff values and performances. In the Romanian population, higher values of Front Kmax, ISV, IVA, KI, IHD, and BAD-D, and lower values of TL and PRC with excellent accuracy (AUCs  >  0.9, where AUC is the area under the curve) were previously reported in individuals with KCN compared to controls [29,30] (Table 1).

Excellent performances have been reported for tomographic and biomechanical measurements of KCN detection in the Romanian population; however, no universal single-cutoff criterion applies across different cohorts. Given the limited evidence available for this population, our study had a two-fold aim. First, to identify tomographic and biomechanical biomarkers that can differentiate between early-stage (mild/moderate) keratoconus (KCN) and more advanced (advanced/severe) KCN, based on ABCD classification. Second, to assess the discriminatory performance of previously reported biomarkers in distinguishing KCN from KCN-free controls. Additionally, the study sought to compare the concordance between the classifications obtained using the cutoffs derived from the current cohort and those previously reported, thereby evaluating the consistency and potential generalizability of these thresholds within the Romanian KCN population.

## 2. Materials and Methods

This study was conducted at the Oculens Clinic in Cluj-Napoca, Romania, in accordance with the latest update (2024) of the Declaration of Helsinki. The study was approved by the Oculens Ethics Committee (approval no. 14/1 December 2023). All procedures complied with ethical standards, ensuring the protection of participants’ rights, safety, and well-being.

### 2.1. Study Methodology

**Figure 1:**
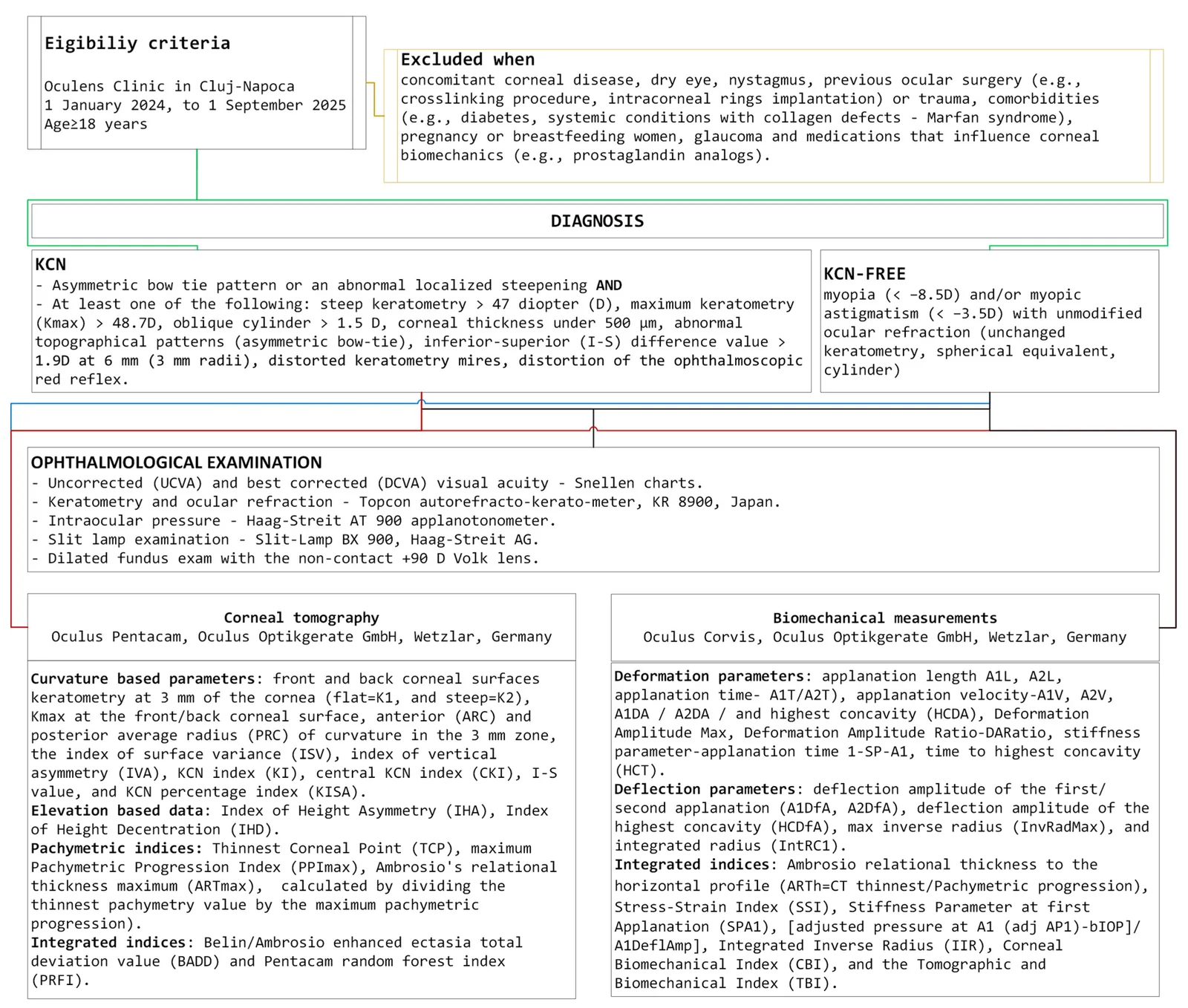
Study design and participant assessment workflow.

The study included individuals who were referred to the Ocules Clinic for healthcare needs related to ocular conditions. The study design and participant assessment workflow are summarized in Figure 1 and described in detail by Nicula et al. [31]. The evaluated participants comprised 44 subjects with KCN and 38 controls with a median age of 26.5 years; a quarter of the participants were younger than 22 years, and another quarter were older than 30 years without a history of previous corneal surgery or concomitant ocular diseases. The participants with KCN were similar to KCN-free individuals in terms of age and sex (*p*-values > 0.05).

The diagnosis of KCN and severity of the disease were established based on current practice guidelines [32,33] and the criteria of the Collaborative Longitudinal Evaluation of Keratoconus study [34]. Corneal tomography (Oculus Pentacam, Oculus Optikgerate GmbH, Wetzlar, Germany; images, captured in 2 s creating a 3D representation including the anterior and posterior cornea [35] and biomechanical measurements (Oculus Corvis, Oculus Optikgerate GmbH, Wetzlar, Germany; 140 horizontal 8 mm frames in 33 ms that allow evaluation of deformation in two dimensions [36]) were made by a single experienced researcher before the fundus examination. The corneal deformation parameters (Corvis ST, Oculus Optikgeräte GmbH, Germany, REF 72100, SN 7210009128150) were calculated based on the dynamic inspection of the corneal response [37].

Corvis ST parameters measure deformation, deflection, and integrated indices [35,38,39]. Ambrosio relational thickness to the horizontal profile (ARTh = CT thinnest/pachymetric progression) [40], stress–strain index—SSI), stiffness parameter at first applanation (SPA1) [((adj AP1)-bIOP)/A1DeflAmp, where adj AP1—adjusted pressure at A1 ) [38], integrated inverse radius (IIR), corneal biomechanical index (CBI) [41], and the tomographic and biomechanical index (TBI) [35,36,38] are also available as Corvis ST integrated.

### 2.2. Statistical Analysis

Only one eye of each participant was included in the analysis. Therefore, all observations were statistically independent, and the patients were considered the unit of analysis throughout the study.

Data was reported considering the type and, whenever applicable, the distribution tested using the Shapiro–Wilk test. Qualitative data were reported as absolute and relative (percentage) frequencies, and comparisons were made using the chi-square family test, respecting assumptions. Quantitative data were reported as mean (standard deviation) when the theoretical normal distribution was not violated; otherwise, median and interquartile range (expressed as [Q1 to Q3], where Q stands for quartile) were reported, and differences between groups were tested using non-parametric methods.

Patients with KCN were grouped based on ABCD classification (A—Anterior Radius of Curvature; B—Posterior Radius of Curvature; C—Thinnest Pachymetry in μm; D—Distance Best Corrected Visual Acuity) [42] as mild or moderate disease (any of four parameters with highest stage as I for mild and II for moderate) and advanced or severe disease (any of four parameters with the highest stage as III for advanced and IV for severe). Two group classification was used to distinguish between early-stage and more advanced forms of keratoconus to facilitate clinically meaningful comparisons while maintaining adequate sample sizes within each severity category. The cutoff values were calculated for all tomography and biomechanical measurements, and the models with AUCs (lower 95% confidence interval bound) higher than 0.9 were considered excellent and good for values between 0.80 and 0.89. The following metrics were reported for excellent models: cutoff value (maximum of Youden index = Se + Sp − 1), sensitivity (Se), specificity (Sp), positive (+LR) and negative (+LR) likelihood ratios, number of true positives (TP), false positives (FP), false negatives (FN), and true negatives (TN). To maximize the performance in distinguishing mild/moderate from advanced/severe disease, a logistic regression analysis was employed. The biomarker with the highest AUC and the one with the highest number of true positives were used as input data in the logistic regression analysis. The probability model was reported along with ORs and ROC curves in training and cross-validation.

The previously reported corneal biomechanical and tomographic indices that proved excellent in distinguishing patients with KCN from KCN-free individuals were tested in ROC analysis to validate their consistency. The performance metrics are reported for the best cutoff value. The classification agreement between the cutoff values obtained in this cohort and those previously reported in the Romanian population for distinguishing between patients with KCN and those who are KCN-free was tested using unweighted Cohen’s kappa. Agreement expressed in percentage, kappa statistic, and associated *p*-value are reported for markers that show good or excellent performance in the ROC analysis. Cohen’s kappa values higher than 0.80 show almost perfect agreement [43].

Statistical analyses were conducted in an exploratory manner using JAMOVI (version 2.7.37.0). To control the increased risk of Type I error associated with testing the 52 biomarkers, a Bonferroni-adjusted significance threshold was adopted (α/m = 0.05/52 = 0.00096). Statistical significance was set at *p* < 0.001.

## 3. Results

### 3.1. Discriminatory Performances of Corneal Tomography and Biomechanical Measurements by Severity

Twenty-three patients had mild (six patients) or moderate (17 patients) KCN while twenty-one had advanced (five patients) or severe (16 patients) disease. The patients were similar in terms of age in the evaluated severity groups (mild/moderate vs. advanced/severe) (Table 2). Corneal tomography and biomechanical measurements showed distinct patterns and performances according to disease severity (Table 2 and Table 3).

**Figure 2:**
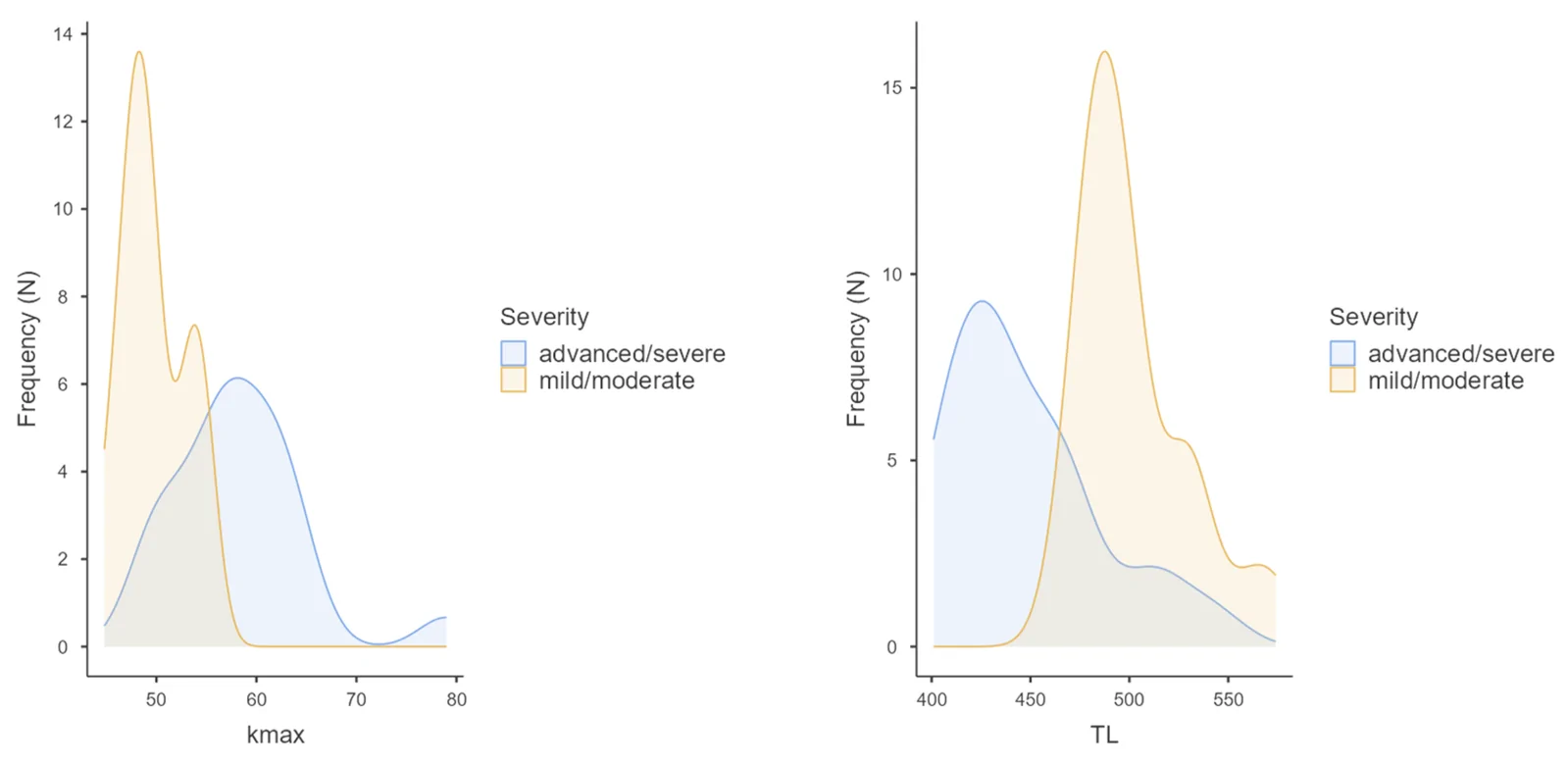
Distribution of Kmax and TL (thinnest location) by severity group.

Kmax showed the greatest performance in terms of AUC, with excellent discriminatory ability in the identification of patients with mild/moderate KCN rather than advanced/severe KCN. The distribution of values by severity class is shown in Figure 2.

**Figure 3:**
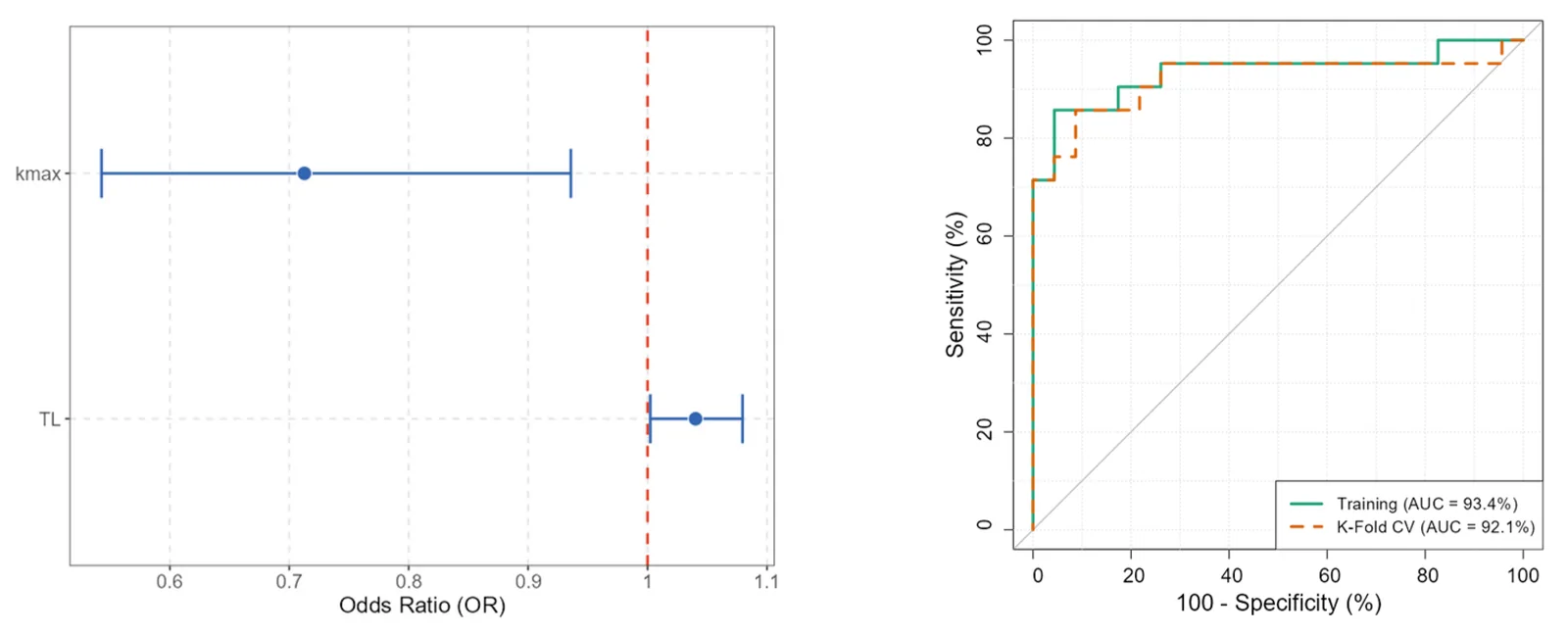
Forest plot of odds ratios: logistic regression model (**left-hand panel**) and model performance in training and cross-validation (CV) (**right-hand panel**).

Combining Kmax with TL in a regression model, with patients with mild/moderate disease as the reference, increased discrimination accuracy (Table 4, Figure 3). The regression model classified three patients with advanced/severe KCN as mild/moderate (false negative) and one patient with mild/moderate as advanced/severe (false positive).

### 3.2. Keratoconus vs. Keratoconus-Free

Forty-four patients with KCN and 38 KCN-free individuals were included in this analysis. Individuals with KCN differed from KCN-free individuals in both Scheimpflug tomography (Pentacam) and Corvis ST biomechanical parameters (Table 5), except for k1p-back, A1L, and RMS-HOA. Individuals in the KCN-free group had normal corneal tomography.

The discriminatory abilities of the previously reported indices, except for KI and BAD-D which proved to be overfitting, were retained in the current cohort, and all exhibited excellent or good discriminatory performances (Table 6).

**Figure 4:**
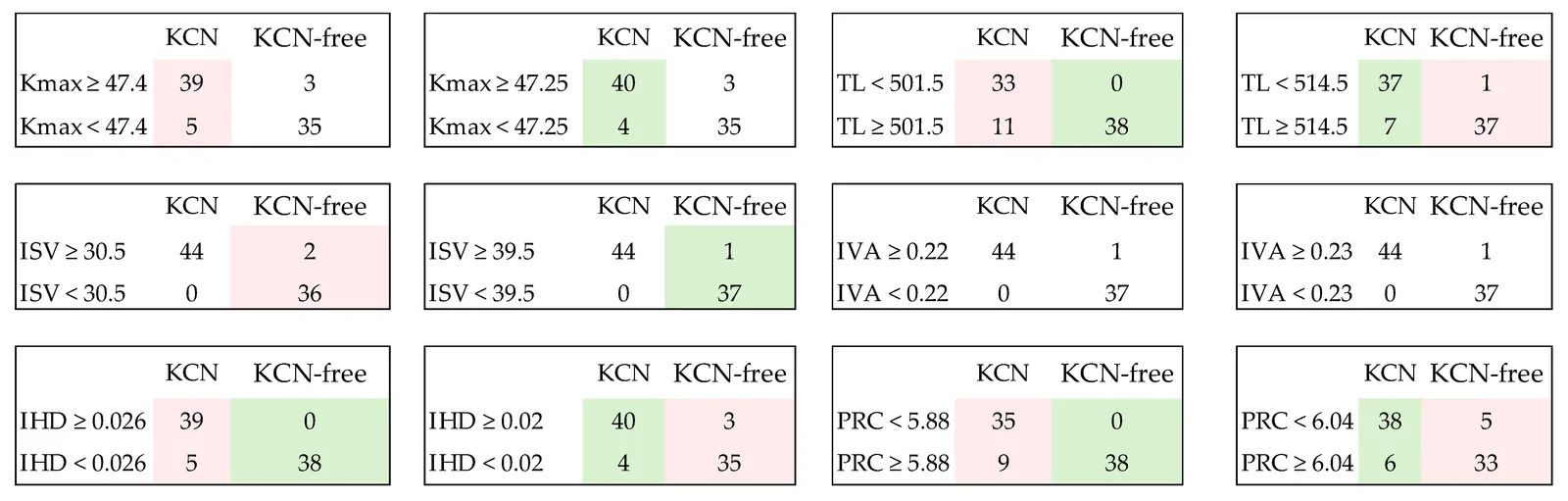
Matrix of classification for distinguishing KCN from KCN-free eyes in the current and previous cohorts. Each panel shows a 2 × 2 contingency table based on the indicated biomarker cutoff value. Numbers represent the count of eyes classified into the KCN and KCN-free groups according to the reported cutoff values. Green shading indicates better classification, red shading indicates inferior classification, and no shading indicates no differences in classification. The evaluated biomarkers included maximum keratometry (Kmax), thinnest corneal thickness (TL), index of surface variance (ISV), index of vertical asymmetry (IVA), index of height decentration (IHD), and posterior radius of curvature (PRC). Thresholds were derived separately for the current and previous cohorts.

The values of true-positive, false-positive, false-negative, and true-negative cases were identical for IVA and changed slightly for the other evaluated markers (Figure 4) when the currently obtained cutoff values were compared with previously reported cutoff values (Figure 4).

**Figure 5:**
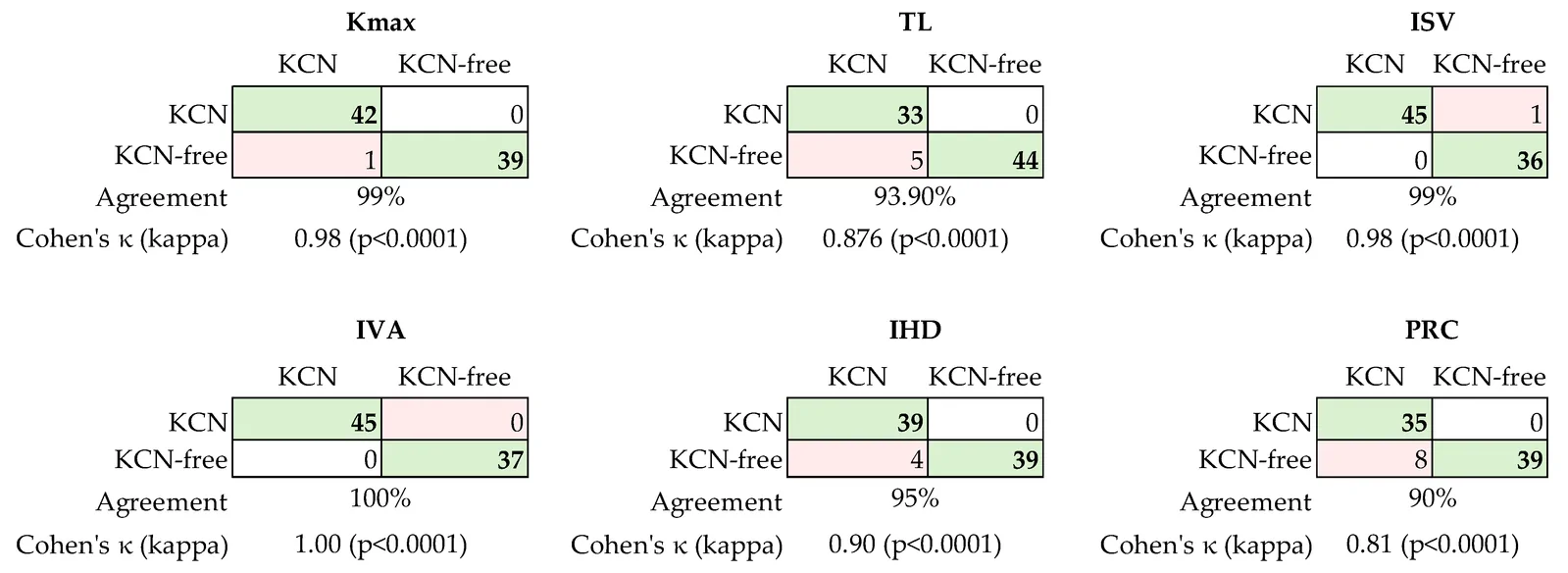
Confusion matrices illustrating the agreement between the current cutoff values (rows) and the classification were generated using previously reported cutoff values (columns). The green cells represent correctly classified cases (true-positive and true-negative), whereas the pink cells represent differences in classification. The numbers in the cells indicate the number of eyes in each category. The evaluated biomarkers included maximum keratometry (Kmax), thinnest location (TL), index of surface variance (ISV), index of vertical asymmetry (IVA), index of height decentration (IHD), and posterior radius of curvature (PRC). Thresholds were derived separately for the current and previous cohorts.

A high diagnostic agreement was observed with two cutoff values for the evaluated biomarkers, characterized by a large number of correctly classified KCN and KCN-free eyes and a low number of false-positive and false-negative classifications (Figure 5).

## 4. Discussion

The results of our study support the robustness of tomographic and biomechanical biomarkers for staging keratoconus severity in a Romanian population and confirm the diagnostic utility of previously reported indices for keratoconus detection. Among the evaluated parameters, Kmax demonstrated the highest ability to discriminate between early-stage and advanced keratoconus, achieving excellent diagnostic performance and supporting its role as a valuable marker of disease severity. The thinnest corneal location (TL) was also strongly associated with disease severity, showing high classification accuracy and a low rate of false-negative results, highlighting its potential clinical relevance in identifying advanced diseases. However, given the cross-sectional design of the study, these findings reflect associations with disease severity at a single time point and should be evaluated in the context of disease progression or predictive ability regarding future progression. The assessment analysis showed that most previously published cutoff values retained their diagnostic performance in the Romanian cohort, accurately distinguishing patients with KCN from KCN-free controls. The exceptions were KI and BAD-D, which demonstrated evidence of overfitting and lower potential generalizability. Importantly, classifications generated using cohort-specific thresholds showed almost perfect agreement with those obtained using previously reported cutoff values, as reflected by Cohen’s kappa coefficients higher than or equal to 0.8 and raw agreement rates exceeding 90%.

### 4.1. Performance in Distinguishing Disease Severity

Advancements in technology have allowed the evaluation of the posterior cornea and the creation of pachymetry maps using three-dimensional tomographic images, such as rotational Scheimpflug imaging or optical coherence tomography [44]. The Pentacam scan (Oculus Optikgerate GmbH, Wetzlar, Germany) is one of the most popular devices [10] used for the diagnosis of subclinical and clinical forms of KCN and for the evaluation of disease progression.

In our study, Kmax showed the highest performance in terms of AUC, with excellent discriminatory abilities in the identification of patients with mild/moderate KCN compared with advanced/severe KCN (Table 3) and in differentiating between KCN and KCN-free individuals (Table 6). In our study, Kmax and TL differed significantly between the two severity groups (Table 3). Although the interquartile ranges did not overlap, individual Kmax values showed partial overlap between groups (Figure 2). This overlap is reflected in the diagnostic performance of Kmax and TL, indicating that Kmax or TL alone cannot fully discriminate between the keratoconus severity stages. One explanation is the multidimensional nature of the ABCD classification system, which incorporates anterior and posterior corneal curvature, thinnest corneal thickness, and best-corrected visual acuity. Consequently, patients with similar Kmax or TL values may be assigned to different severity categories based on differences in these additional anatomical or functional parameters. Moreover, the overlap observed in some biomechanical parameters likely reflects the heterogeneous pattern of corneal biomechanical changes that characterize keratoconus progression. As expected, combining Kmax and TL increased the discriminatory performance (Table 4 and Figure 3) within the study cohort. The logistic regression model demonstrated promising discriminatory performance within the study cohort. However, in the absence of independent validation, its clinical utility remains to be established and should be confirmed in future studies. Similarly, Rabinowitz and McDonnell [45] revealed that central corneal power was steeper in KCN eyes than in normal eyes, and the mean central power found in KCN eyes was 52.13 (range 43.10–60.20). Moreover, Mihaltz et al. [46] showed that posterior and anterior elevation had the best predictive accuracy, followed by minimal and central pachymetry (AUC = 0.89 and 0.88) between mild/moderate KCN and the controls.

Our study revealed that topographic indices such as TL (AUC = 0.943, Table 3), ISV (AUC = 0.99, Table 3), IVA (AUC = 0.998, Table 3), IHD (AUC = 0.986, Table 3), and PRC (AUC = 0.903, Table 3) showed high abilities to identify KCN and differentiate between severity classes. Similarly, the posterior elevation value is a very sensitive and precise diagnostic index for identifying subtle changes in KCN [47]. However, several controversies exist regarding KCN detection [48]. Hashemi et al. showed that BAD_D, mean keratometry, and third order vertical coma aberration were the best diagnostic indicators for KCN [49]. Mothglah et al. [50] showed that most of the topographic indices, including anterior elevation, CKI (central keratoconus index), KI (keratoconus index), and IHA, have high precision in differentiating clinical KCN from normal eyes. Huseinly et al. [51] revealed that IHD had the highest diagnostic accuracy for KCN (AUC = 0.979), followed by IHA (AUC = 0.884). Others have validated IHD as a more sensitive index than BAD-D for the diagnosis of KCN (AUC = 0.97 vs. 0.89) [52].

Our study showed that pachymetric indices of Pentacam such as ARTh, ART Max, and CCT were also valid indices for the diagnosis of clinical KCN (*p* < 0.0001). Similarly, studies have shown that ART, the ratio between the thinnest point and PPI, is a novel parameter for the diagnosis of KCN [53,54,55] with the highest diagnostic accuracy at a cutoff value of 300–400 µm [56]. Seiler et al. [57] demonstrated the impact of age on corneal biomechanics and differences between normal and keratoconic corneas, but the accuracy of the reported findings is relatively weak and needs further research.

Our results indicate that the parameters that provided a statistically significant differentiation between mild/moderate and severe/advanced KCN cases were anterior elevation (K1, K2, Kmax), posterior elevation (K2p back, Kmeanp bacK), CCT, PPI, ART Max, ARTh, ISV, IVA, IHD, and BAD-D (*p* < 0.0001). Similarly, Du et al. [58] revealed that between the subclinical and moderate stages, the diagnostic efficiency of all tomography indices (posterior and anterior elevation, anterior Kmax, and anterior I-S to posterior I-S, and posterior Kmax) were high (all AUC ≥ 0.9). Moreover, the same study showed that when KCN became severe, the diagnostic efficiency of characteristic indices decreased from the anterior Kmax, posterior Kmax, anterior SimKm, anterior Kmin to posterior elevation, anterior BFS, and anterior elevation, all being high (all AUC > 0.9) [58].

The evolution of the assessment of corneal biomechanics allowed important advances in the diagnosis, staging, and prognosis of KCN [8,10,59]. Vinciguerra et al. described the Corvis biomechanical index (CBI) [41]. The combination of Corvis parameters with tomographic parameter in TBI demonstrated superior accuracy for the detection of ectatic disease or even ectasia susceptibility [8].

Our study showed that CBI and TBI had high diagnostic accuracy for distinguishing KCN from normal eyes (*p* < 0.0001, Table 6). Despite affecting corneal biomechanical factors, CBI and TBI are created to diagnose KCN rather than detect progression [8]. Furthermore, Yang et al. showed that DA MAX, A1T, A1V, A2V, radius, A1DA, HCDA, A2DA, A1DLA, HCDLA, A2DLA, DLAML, A1DLAr, A1dArcL, and A2dArcL were significantly different between KCN and normal eyes [60]. The same study revealed that the Max Inverse Radius, DA Ratio Max (2 mm), Pachy Slope, DA Ratio Max (1 mm), Integrated Radius, and CBI in KC eyes were higher than in normal eyes, while the ARTh and SP-A1 were lower than those of normal eyes (all *p* < 0.05) [60]. Moreover, most studies also found that TBI has a higher diagnostic ability to distinguish KCN [61], even in different stages of KCN [7]. Zhang et al. [11] showed that TBI not only had the highest AUC value (CKC vs. normal, 1.000; FFKC vs. normal, 0.885), but it was also the single parameter that proved its efficiency in KCN diagnostic.

### 4.2. Consistency of Cutoff Values

The present study demonstrated clear and consistent differences in both corneal tomographic and biomechanical characteristics between keratoconus (KCN) and KCN-free eyes (Table 5 and Table 6). Our results confirm that KCN is associated with substantial alterations in the corneal shape, thickness distribution, elevation profile, and biomechanical behavior. These findings support the current understanding that biomechanical alterations occur alongside, and potentially before, the detectable tomographic changes.

A major strength of our study was the assessment of previously reported diagnostic cutoff values. Most evaluated biomarkers maintained their discriminatory performance in the Romanian cohort (Figure 4 and Figure 5), indicating that their diagnostic utility is not limited to the populations in which they were originally developed.

Among all the evaluated indices, ISV and IVA demonstrated the highest performance, both achieving an AUC of 0.998 with perfect sensitivity (100%) and specificity of 97.4% (Table 6). Only one KCN-free eye was misclassified, whereas all KCN eyes were correctly identified. Such results indicate near-perfect diagnostic performance and support the robustness of these topometric indices. The values of IHD showed outstanding performance (AUC = 0.986, Table 6), whereas Kmax retained excellent discrimination (AUC = 0.962, Table 6), confirming its role as a simple and potentially clinically applicable marker for keratoconus detection. The thinnest location indices, such as TL (AUC = 0.943, Table 6) and PRC (AUC = 0.903, Table 6), also demonstrated excellent to good diagnostic accuracy. The high positive likelihood ratios observed for Kmax, ISV, IVA, IHD, and TL (Table 6) suggest that positive test results increase the probability of keratoconus, whereas the very low negative likelihood ratios indicate strong rule-out capabilities.

An important objective of our study was to assess whether the thresholds reported in previously investigated Romanian cohorts retained consistency. The findings indicate excellent preservation of classification performance across cohorts (Figure 4 and Figure 5). The confusion matrices demonstrate only minor differences in true-positive, false-positive, false-negative, and true-negative classifications when current and previously reported cutoffs were compared. Furthermore, the high agreement shown in Figure 5 indicates that biomarker classifications remain highly stable despite variations in the evaluated cohorts. This suggests that ISV, IVA, IHD, Kmax, TL, and PRC may represent reliable biomarkers with diagnostic performance that appear to be consistent across evaluated cohorts of the same population. Nevertheless, in the current study, KI and BAD-D appeared to exhibit evidence of overfitting. Although these indices differed significantly between groups, their previously reported thresholds were not reproduced in the current cohort. This finding highlights the variability within cohorts of the same population and the need for studies on independent populations to formally validate these thresholds and implement them in current practice.

### 4.3. Study Limitations

Several limitations should be considered when interpreting the reported results. First, the sample size was relatively modest, with only 44 patients with KCN and 38 controls without an external cohort for validation. Although statistically significant differences were observed for most parameters, the small sample size may have resulted in unstable estimates of sensitivity, specificity, and cutoff values. Interpretation of the very high AUC values warrants caution, as the thresholds were derived and tested in the same study population, potentially resulting in overestimated diagnostic performance. Second, this was a single-center study conducted on a Romanian population. Consequently, the results may not be fully generalizable to populations with different ethnic, genetic, environmental, or healthcare characteristics. Third, the cross-sectional design precluded the evaluation of disease progression. Therefore, while our study identified parameters associated with keratoconus, it could not determine which biomarkers are most useful for predicting future progression. Fourth, multiple tomographic and biomechanical parameters were evaluated simultaneously. Although this provides a comprehensive assessment, it increases the risk of identifying statistically significant findings because of multiple testing. To minimize this, we applied a Bonferroni adjustment for the significance level. Fifth, some variables, particularly aberration-based indices, may have been influenced by measurement variability and the relatively limited sample size, potentially reducing their apparent discriminatory ability.

## 5. Conclusions

The findings reported in this study confirm that keratoconus is associated with profound tomographic and biomechanical abnormalities that can be reliably detected using the Pentacam and Corvis ST parameters. ISV, IVA, IHD, Kmax, TL, and PRC demonstrated excellent diagnostic performance and maintained their effectiveness when previously established cutoff values were applied to this cohort. The high level of agreement between cohort-specific and previously reported thresholds suggests the robustness and potential generalizability of these biomarkers in similar populations. While our findings support the potential diagnostic utility of these parameters for keratoconus detection and severity assessment, the results should be considered preliminary, given the single-center design, relatively small sample size, and lack of external validation. Larger multicenter and longitudinal studies are needed to confirm these findings, establish their generalizability across diverse populations and disease stages, and determine their suitability for routine clinical screening and diagnosis of this disease.

## Figures and Tables

**Table 1:** Corneal shape and biomechanics markers for keratoconus in the Romanian population.

Nicula et al. 2024 [29] *			Nicula et al. 2022 [30] **		
Marker	AUC [95%CI]	Cutoff	Marker	AUC [95%CI]	Cutoff
Front Kmax	0.978 [0.963 to 0.994]	47.4	Front Kmax	0.981a [0.963 to 0.998]	0.916
ISV	0.998 [0.995 to 1]	30.5	ISV	0.999 [0.998 to 1]	34.5
IVA	0.998 [0.995 to 1]	0.22	KISA	0.999 [0.998 to 1]	66.042
KI	0.991 [0.979 to 1]	1.065	KI	0.989 [0.972 to 1]	1.065
IHD	0.993 [0.986 to 0.999]	0.026	IHD	0.984 [0.965 to 1]	0.026
BAD-D	0.985 [0.964 to 1]	1.395	BAD-D	0.987 [0.97 to 1]	2.63
TL	0.958 [0.935 to 0.981]	↓ 510.5	RMS-HOA	0.997 [0.992 to 1]	3.011
PRC	0.962 [0.935 to 0.989]	↓ 5.88	PRC (mm)	0.994 [0.983 to 1]	↓ 5.825
			ART max	0.983 [0.961 to 1]	345.5
			TP (μm)	0.98 [0.963 to 0.997]	502

* n = 27 patients with KCN, 73 eyes vs. 41 individuals KCN free, 70 eyes; ** n = 52 patients with KCN, 95 eyes vs. 55 controls, 105 eyes; ART Max = maximum Ambrósio relational (μm); TP = thinnest point (μm); IHD = index of height decentration; ↓ lower values indicate the presence of disease. ISV = index of surface variance; IVA = index of vertical asymmetry; KI = keratoconus index; BAD-D = Belin/Ambrosio enhanced ectasia total deviation value; TL = thinnest location (μm); PRC = posterior radial curvature (mm); RMS-HOA = root mean square of higher-order aberrations.

**Table 2:** Characteristics of the cohort by KCN severity.

	Mild/Moderate (n = 23)	Advanced/Severe (n = 21)	*p*-Value
Demographics			
Age, years	27 [19.5 to 31]	25 [20 to 32]	0.7143
Men, n (%)	14 (47)	16 (53)	0.2482
Curvature-based indices			
K1a (D)	43.2 [43 to 44.2]	47.2 [43.8 to 49.8]	0.0008
K2a (D)	46.2 [45.4 to 47.4]	50.5 [48.8 to 53.1]	0.0003
kmax (D)	48.9 [47.4 to 53]	58.1 [54.7 to 62.1]	<0.0001
K1p-back (D)	−6.1 [−6.3 to −6]	−6.9 [−7.5 to −6.1]	0.002
K2p-back (D)	−6.8 [−7 to −6.6]	−7.7 [−8.1 to −7.6]	<0.0001
Kmeanp (D)	−6.4 [−6.6 to −6.4]	−7.2 [−8 to −6.8]	<0.0001
Thickness and pachymetric indices			
TL	492 [482 to 515.5]	434 [423 to 468]	<0.0001
CCT	506 [488 to 531.5]	464 [432 to 476]	<0.0001
PI	1.4 [1.3 to 1.6]	1.9 [1.8 to 2.2]	0.0002
ARTMax	228 [203 to 249.5]	175 [138 to 203]	0.0004
ARTh	237.2 [153.5 to 436.3]	282.8 [145 to 326.9]	0.7244
Vol C	58.2 [55.9 to 60.5]	55.6 [54.6 to 56.8]	0.0303
Critical topometric indices			
ISV	58 [49 to 72]	93 [76 to 113]	0.0004
IVA	0.6 [0.5 to 0.91]	1.06 [0.87 to 1.26]	0.0007
KI	1.12 [1.11 to 1.21]	1.28 [1.14 to 1.33]	0.0147
CKI	1.04 [1.01 to 1.06]	1.05 [1.05 to 1.08]	0.0301
KISA (%)	228.4 [158.1 to 373.9]	397.3 [228.4 to 1729]	0.0034
InegR	9.5 [8.1 to 11.7]	13.2 [10.9 to 14.5]	0.0073
PRFI	0.98 [0.94 to 0.99]	1 [0.96 to 1]	0.0624
Elevation and shape parameters			
ARC (mm)	7.2 [7.1 to 7.6]	6.8 [6.3 to 7.5]	0.1176
PRC (mm)	5.6 [5.2 to 5.9]	5.1 [4.8 to 5.8]	0.0383
I-S (D)	4.7 [3.7 to 5.9]	5.9 [4.7 to 8.8]	0.0295
Integrated Radius	11.1 [8.4 to 13.1]	11 [10.6 to 14.9]	0.3178
IHD	0.06 [0.05 to 0.09]	0.13 [0.07 to 0.19]	0.0015
IHA	37.4 [33.1 to 56]	32.5 [31.8 to 58]	0.5091
DaRatio	4.7 [4.3 to 5.2]	5.5 [4.9 to 6.3]	0.0054
BAD	5 [4.7 to 7.2]	8.3 [6.7 to 10.7]	0.0031
BAD-D	5.5 [5 to 6.5]	9.4 [7.4 to 10.7]	<0.0001
Aberrometry based indices			
RMSTotal	191.5 [179.8 to 205.9]	193.3 [187 to 206.8]	0.4242
RMS-HOA	7.5 [6.1 to 8.5]	7.6 [6.7 to 8.2]	0.6552
BCVA	0.8 [0.5 to 0.9]	0.4 [0.2 to 0.7]	0.0004
Biomechanical indices from Corvis			
CBI	0.9 [0.8 to 0.9]	1 [0.9 to 1]	0.0125
A1L (mm)	2.16 [1.96 to 2.35]	1.87 [1.48 to 2.01]	0.0215
A2L (mm)	1.87 [1.51 to 1.89]	1.76 [1.27 to 1.84]	0.2633
A1Time	7.2 [6.9 to 7.4]	7 [6.7 to 7.1]	0.0862
A2Time	21.9 [21.5 to 22.1]	22 [21.9 to 22.1]	0.0906
A1Velocity (m/s)	0.15 [0.13 to 0.17]	0.16 [0.15 to 0.17]	0.5409
A2Velocity (m/s)	−0.3 [−0.3 to −0.3]	−0.3 [−0.3 to −0.3]	0.0798
SPA1	76.4 [69.2 to 102.3]	76.8 [55.9 to 84.5]	0.1728
DARatioMax2 (mm)	4.9 [4.4 to 5.9]	5.4 [4.9 to 6]	0.2129
DARatioMax1 (mm)	1.67 [1.59 to 1.74]	1.69 [1.66 to 1.78]	0.1656
HCDefAmp	1.05 [0.97 to 1.17]	1.1 [1.03 to 1.21]	0.1764
A1DefAmp	0.14 [0.13 to 0.15]	0.14 [0.13 to 0.14]	0.2791
A2DefAmp	0.4 [0.35 to 0.41]	0.4 [0.35 to 0.41]	>0.9999
DefAmpMax	1.05 [0.97 to 1.17]	1.1 [1.03 to 1.21]	0.1881
A1DeflectAmp	0.1 [0.09 to 0.11]	0.1 [0.1 to 0.11]	0.9344
A2DeflectAmp	0.12 [0.11 to 0.12]	0.12 [0.11 to 0.13]	0.5329
InvRadMaxIntRC1	0.18 [0.14 to 0.19]	0.18 [0.15 to 0.19]	0.4401

Data are reported as median [Q1 to Q3], where Q is the value of the first (Q1) and third (Q3) quartiles, except for sex, when absolute frequency (%) is reported. Comparisons were made using the Mann–Whitney test, except for sex, when the chi-squared test was applied.

**Table 3:** ROC analysis for discrimination between mild/moderate and advanced/severe KCN.

	Cutoff	AUC [95%CI]	*p*-Value	Se	Sp	Acc	+LR	−LR	TP	FP	FN	TN
K1a	43.70	0.796 [0.649 to 0.943]	<0.0001	80.95 [58.09 to 94.55]	69.57 [47.08 to 86.79]	75.0 [59.66 to 86.61]	2.66	0.27	17	7	4	16
K2a	48.50	0.817 [0.670 to 0.963]	<0.0001	76.19 [52.83 to 91.78]	86.96 [66.41 to 97.22]	81.82 [67.29 to 91.81]	5.84	0.27	16	3	5	20
Kmax	54.15	0.908 [0.824 to 0.992]	<0.0001	76.19 [52.83 to 91.78]	86.96 [66.41 to 97.22]	81.82 [67.29 to 91.81]	5.84	0.27	16	3	2	20
K1p-back	↓ −6.45	0.771 [0.619 to 0.923]	0.0002	66.67 [43.03 to 85.41]	95.65 [78.05 to 99.89]	81.82 [67.29 to 91.81]	15.33	0.35	14	1	7	22
K2p-back	↓ −7.25	0.853 [0.725 to 0.981]	<0.0001	80.95 [58.09 to 94.55]	86.96 [66.41 to 97.22]	81.09 [69.93 to 93.36]	6.21	0.22	17	3	4	20
Kmeanp back	↓ −6.75	0.874 [0.759 to 0.989]	<0.0001	80.85 [58.09 to 94.55]	86.96 [66.41 to 97.22]	81.09 [69.93 to 93.36]	6.21	0.22	17	3	4	20
Vol C	56.95	0.692 [0.527 to 0.856]	0.0111	76.19 [52.83 to 91.78]	65.22 [42.73 to 83.62]	70.75 [54.80 to 83.24]	2.19	0.37	16	8	5	15
TL	↓ 475.00	0.880 [0.755 to 1.0]	<0.0001	85.71 [63.66 to 96.95]	95.65 [78.05 to 99.89]	90.91 [78.33 to 97.47]	19.71	0.15	18	1	3	22
CCT	479.50	0.872 [0.745 to 0.998]	<0.0001	80.95 [58.09 to 91.55]	100 [85.15 to 100]	90.91 [78.33 to 97.47]	inf	0.19	17	0	4	23
PI	1.77	0.829 [0.695 to 0.963]	<0.0001	80.95 [58.09 to 94.55]	86.96 [66.41 to 97.22]	84.09 [69.93 to 93.36]	6.21	0.22	17	3	4	20
ARTMax	↓ 182.50	0.812 [0.677 to 0.946]	<0.0001	71.43 [47.82 to 88.72]	91.30 [71.96 to 98.93]	81.82 [67.29 to 91.81]	8.21	0.31	15	2	6	21
ISV	75.00	0.811 [0.675 to 0.946]	<0.0001	76.19 [52.83 to 91.78]	78.26 [56.30 to 92.54]	77.27 [62.16 to 88.53]	3.50	0.30	16	5	5	18
IVA	0.93	0.799 [0.665 to 0.933]	<0.0001	71.43 [47.82 to 88.72]	78.26 [56.30 to 92.54]	75.00 [59.66 to 86.81]	3.29	0.37	15	5	6	18
KI	1.13	0.715 [0.561 to 0.869]	0.0031	80.95 [58.09 to 94.55]	56.52 [34.49 to 76.81]	68.18 [52.42 to 81.39]	1.86	0.34	17	10	4	13
IHA	↓ 34.20	0.559 [0.375 to 0.734]	0.2642	66.67 [43.03 to 85.41]	60.87 [38.54 to 80.29]	63.64 [47.77 to 77.59]	1.70	0.55	14	9	7	14
IHD	0.08	0.780 [0.638 to 0.921]	<0.0001	71.43 [47.82 to 88.72]	69.57 [47.08 to 86.79]	70.45 [54.80 to 82.34]	2.35	0.41	15	7	6	16
KISA	275.82	0.759 [0.617 to 0.900]	0.0002	71.43 [47.82 to 88.72]	69.57 [47.08 to 86.79]	70.45 [54.80 to 82.34]	2.35	0.41	15	7	6	16
ARC	↓ 6.83	639 [0.464 to 0.814]	0.600	57.14 [34.02 to 78.18]	86.96 [66.41 to 97.22]	72.73 [57.21 to 85.04]	4.38	0.49	12	3	9	20
PRC	↓ 5.22	0.683 [0.509 to 0.587]	0.0195	61.90 [38.44 to 81.89]	82.61 [61.22 to 95.05]	72.73 [57.21 to 85.04]	3.56	0.46	13	4	8	19
IS	5.71	0.693 [0.534 to 0.851]	0.0086	52.38 [29.78 to 74.29]	69.57 [47.08 to 86.79]	61.36 [45.50 to 75.64]	1.72	0.68	11	7	10	16
SP1	↓ 83.95	0.772 [0.626 to 0.918]	0.0001	76.19 [52.83 to 91.78]	78.26 [56.30 to 92.54]	77.27 [62.19 to 88.53]	3.50	0.30	16	5	5	18
InegR	12.05	0.737 [0.586 to 0.889]	0.0011	61.90 [38.44 to 81.89]	82.61 [61.22 to 95.05]	72.73 [57.21 to 85.04]	3.56	0.46	13	4	8	19
DaRatio	5.20	0.745 [0.594 to 0.897]	0.0007	66.67 [43.03 to 85.41]	73.91 [51.59 to 89.77]	70.45 [54.80 to 83.24]	2.56	0.45	14	6	7	17
ARTh	↓ 219.80	0.783 [0.644 to 0.921]	<0.0001	71.43 [47.82 to 88.72]	86.96 [66.41 to 97.22]	79.55 [64.70 to 90.20]	5.48	0.33	15	3	6	20
BAD	6.44	0.761 [0.611 to 0.911]	0.0003	80.95 [58.09 to 94.55]	69.57 [47.08 to 86.79]	75.00 [59.66 to 86.81]	2.66	0.27	17	7	4	16
PRFI	0.99	0.660 [0.501 to 0.820]	0.0243	61.90 [38.44 to 81.89]	56.52 [34.49 to 76.81]	59.09 [43.25 to 73.66]	1.42	0.67	13	10	8	13
CBI	0.97	0.719 [0.561 to 0.878]	0.0033	61.90 [38.44 to 81.89]	82.61 [61.22 to 95.05]	72.73 [57.21 to 85.04]	3.56	0.46	13	4	8	19
BAD-D	7.00	0.856 [0.745 to 0.968]	<0.0001	85.71 [63.66 to 96.95]	78.26 [56.30 to 92.54]	81.82 [67.29 to 91.81]	3.94	0.18	18	5	3	18
A1L	↓ 2.02	0.703 [0.543 to 0.863]	0.0065	76.19 [82.83 to 91.78]	69.57 [47.08 to 86.79]	72.73 [57.21 to 85.04]	2.50	0.34	16	7	5	16
A2L	↓ 1.86	0.599 [0.423 to 0.776]	0.1347	80.95 [58.09 to 94.55]	52.17 [30.59 to 73.18]	65.91 [50.08 to 79.51]	1.69	0.37	17	11	4	12
A1Velocity	0.16	0.555 [0.376 to 0.733]	0.2733	57.14 [34.02 to 78.18]	60.87 [38.54 to 80.29]	59.09 [43.25 to 73.66]	1.46	0.70	12	9	9	14
A2Velocity	↓ −0.29	0.655 [0.491 to 0.819]	0.0318	52.38 [29.78 to 74.29]	73.91 [51.59 to 89.77]	63.64 [47.77 to 77.59]	2.01	0.64	11	6	10	17
SPA1	76.62	0.379 [0.208 to 0.550]	0.9176	52.38 [29.78 to 74.29]	52.17 [30.59 to 73.18]	52.27 [36.69 to 67.54]	1.10	0.91	11	11	10	12
DARatioMax2	4.89	0.611 [0.440 to 0.781]	0.1012	76.19 [52.83 to 91.78]	52.17 [30.59 to 73.18]	63.64 [47.77 to 77.59]	1.59	0.46	16	11	5	12
DARatioMax1	1.68	0.623 [0.455 to 0.792]	0.0761	66.67 [43.03 to 85.41]	56.52 [34.49 to 76.81]	61.36 [45.50 to 75.64]	1.53	0.59	14	10	7	13
HCDefAmp	1.06	0.620 [0.451 to 0.789]	0.0820	61.90 [38.44 to 81.89]	52.17 [30.59 to 73.18]	56.82 [41.03 to 71.65]	1.29	0.73	13	11	8	12
Integrated Radius	↓ 11.06	0.411 [0.234 to 0.588]	0.8378	57.14 [34.02 to 78.18]	52.17 [30.59 to 73.18]	54.55 [38.85 to 69.61]	1.19	0.82	12	11	9	12
ARTh2	272.22	0.468 [0.292 to 0.644]	0.6393	52.38 [29.78 to 74.29]	56.52 [34.49 to 76.81]	54.55 [38.85 to 69.61]	1.20	0.84	11	10	10	13
RMS Total	186.28	0.571 [0.396 to 0.747]	0.2120	76.19 [52.83 to 91.78]	39.13 [19.71 to 61.46]	56.82 [41.03 to 71.65]	1.25	0.61	16	14	5	9
RMS-HOA	↓ 7.57	0.540 [0.365 to 0.716]	0.3262	52.38 [29.78 to 74.29]	39.13 [19.71 to 61.46]	45.45 [30.39 to 61.15]	0.86	1.22	11	14	10	9

AUC, Se, Sp, Acc are reported as point estimators with 95% confidence interval. Square brackets [ ] indicate the confidence interval, with the lower and upper limits included in the reported range. ↓ symbol—the lower values indicate the presence of advanced/severe KCN.

**Table 4:** Logistic regression model to distinguish advanced/severe from mild/moderate KCN.

Predictor	Estimate (B)	Std. Error	*p*-Value	Odds Ratio (OR)
(Intercept)	0.76	11.80	0.9484	
Kmax	0.34	0.14	0.0148	1.40 [1.07 to 1.84]
TL	−0.04	0.02	0.0373	0.96 [0.93 to 1.0]

TL = thinnest location.

**Table 5:** Tomography (Pentacam) and biomechanical parameters (Corvis ST) by group.

	KCN (n = 44)	KCN-Free (n = 38)	*p*-Value
Curvature-based indices			
K1a (D)	43.8 [43 to 46.9]	43 [41.5 to 44.2]	0.0004
K2a (D)	47.8 [45.6 to 50.6]	45 [43.5 to 45.9]	<0.0001
Kmax (D)	53 [48.9 to 56.9]	45.4 [44.1 to 46.4]	<0.0001
K1p-back (D)	−6.3 [−6.8 to −6]	−6.3 [−6.4 to −6]	0.0872
K2p-back (D)	−7.1 [−7.7 to −6.6]	−6.6 [−6.7 to −6.5]	<0.0001
Kmean p (D)	−6.7 [−7.2 to −6.4]	−6.4 [−6.6 to −6.2]	0.0002
Thickness and pachymetric indices			
TL (um)	478 [434 to 501.3]	563.5 [531.3 to 576.8]	<0.0001
CCT (um)	487 [466.3 to 509.8]	548 [534.5 to 568]	<0.0001
PI	1.66 [1.36 to 1.96]	0.04 [0.03 to 0.1]	<0.0001
ARTMax	205.5 [172 to 236]	456 [443 to 467]	<0.0001
ARTh	244.1 [167.5 to 337.2]	474.9 [422.1 to 558]	<0.0001
VolC	56.5 [55.4 to 59.7]	62.9 [60.9 to 64.4]	<0.0001
Critical topometric indices			
ISV	74 [56.3 to 96.3]	16.5 [13 to 22.5]	<0.0001
IVA (mm)	0.9 [0.56 to 1.07]	0.1 [0.07 to 0.14]	<0.0001
KI	1.19 [1.11 to 1.32]	1.02 [0.99 to 1.03]	<0.0001
CKI	1.05 [1.02 to 1.07]	1.01 [1 to 1.01]	<0.0001
KISA (%)	275.8 [183.2 to 1007]	3.7 [1.8 to 5.2]	<0.0001
Elevation and shape parameters			
ARC (mm)	7.2 [6.6 to 7.5]	7.7 [7.5 to 7.9]	<0.0001
PRC (mm)	5.5 [5.1 to 5.9]	6.3 [6.2 to 6.4]	<0.0001
I-S (D)	5.3 [4.1 to 6.9]	0.1 [−0.5 to 0.3]	<0.0001
Integrated R (mm)	10.9 [9 to 13.8]	7.8 [7.4 to 8.4]	<0.0001
IHD	0.07 [0.06 to 0.15]	0.01 [0.01 to 0.01]	<0.0001
IHA	33.8 [32.5 to 56.5]	4.4 [2.3 to 7]	<0.0001
DaRatio	4.9 [4.5 to 5.9]	4.2 [3.9 to 4.4]	<0.0001
BAD	6.7 [4.9 to 8.6]	1.4 [1.3 to 1.5]	<0.0001
BAD-D	7.3 [5.2 to 9.6]	1 [0.4 to 1.4]	<0.0001
Aberrometry based indices			
RMSTotal	192.4 [183.6 to 206.6]	175.6 [171.1 to 180.2]	<0.0001
RMS-HOA	7.5 [6.3 to 8.2]	1.9 [1.7 to 2.1]	0.3264
BCVA	0.65 [0.4 to 0.9]	0.7 [0.53 to 0.9]	<0.0001
Biomechanical indices from Corvis			
CBI	0.91 [0.85 to 0.99]	0.03 [0.01 to 0.09]	<0.0001
TBI	1 [1 to 1]	0.22 [0.13 to 0.23]	<0.0001
A1L (mm)	2.01 [1.58 to 2.31]	0.13 [0.13 to 0.14]	0.0927
A2L (mm)	1.76 [1.34 to 1.89]	0.34 [0.29 to 0.36]	<0.0001
A1Time	7 [6.8 to 7.2]	7.5 [7.3 to 7.6]	<0.0001
A2Time	21.9 [21.8 to 22.1]	21.6 [21.5 to 21.9]	<0.0001
A1Velocity (m/s)	0.16 [0.13 to 0.17]	0.12 [0.12 to 0.13]	<0.0001
A2Velocity (m/s)	−0.27 [−0.31 to −0.26]	−0.26 [−0.28 to −0.25]	0.0071
SPA1(mm/mmHg)	76.6 [63 to 89.3]	115.4 [107.3 to 120.6]	<0.0001
DARatioMax2 (mm)	5.3 [4.6 to 6]	1.6 [1.6 to 1.6]	<0.0001
DARatioMax1 (mm)	1.69 [1.64 to 1.78]	4.27 [4 to 4.59]	<0.0001
HC PK (mm)	1.08 [1 to 1.18]	3.06 [2.82 to 3.36]	<0.0001
A1DefAmp	0.14 [0.13 to 0.14]	0.13 [0.13 to 0.14]	0.0146
A2DefAmp	0.4 [0.35 to 0.42]	0.34 [0.29 to 0.38]	<0.0001
DefAmpMax	1.08 [1 to 1.18]	0.85 [0.79 to 0.91]	0.0022
A1DeflectAmp	0.1 [0.09 to 0.11]	2.26 [2.21 to 2.34]	<0.0001
A2DeflectAmp	0.12 [0.11 to 0.12]	0.11 [0.1 to 0.11]	<0.0001
InvRadMaxIntRC1	0.18 [0.14 to 0.19]	0.17 [0.15 to 0.17]	<0.0001
Integrated Radius	11.01 [9.13 to 13.2]	7.77 [7.42 to 8.36]	<0.0001

Data are expressed as median [Q1 to Q3] where Q is the value of the first and third quartiles. Comparisons were made using the Mann–Whitney test.

**Table 6:** Discriminatory performances of the evaluated indices: KCN vs. KCN-free (reference).

	Cutoff	AUC [95%CI]	*p*-Value	Se	Sp	Acc	+LR	−LR	TP	FP	FN	TN
Kmax	↑ 47.25	0.962 [0.922 to 1.0]	<0.0001	0.91 [0.78 to 0.97]	0.92 [0.79 to 0.98]	0.91 [0.83 to 0.97	11.52	0.10	40	3	4	35
ISV	↑ 39.50	0.998 [0.994 to 1.0]	<0.0001	1 [0.92 to 1]	0.97 [0.86 to 1]	0.99 [0.93 to 1]	38.0	<0.001	44	1	0	37
IVA	↑ 0.23	0.998 [0.992 to 1]	<0.0001	1 [0.92 to 1]	0.97 [0.86 to 1]	0.99 [0.93 to 1]	38.0	<0.001	44	1	0	37
IHD	↑ 0.02	0.986 [0.969 to 1]	<0.0001	0.91 [0.78 to 0.97]	0.92 [0.79 to 0.98]	0.91 [0.83 to 0.97]	11.52	0.10	40	3	4	35
TL	↓ 514.50	0.943 [0.90 to 0.99]	<0.0001	0.84 [0.70 to 0.93]	0.97 [0.86 to 0.99]	0.90 [0.82 to 0.96]	31.96	0.16	37	1	7	37
PRC	↓ 6.04	0.903 [0.83 to 0.98]	<0.0001	0.86 [0.73 to 0.95]	0.87 [0.72 to 0.96]	0.87 [0.77 to 93]	6.56	0.16	38	5	6	33

*p*-values belong to the DeLong test; Se = sensitivity; Sp = specificity; Acc = accuracy; +LR = positive likelihood ratio; −LR = negative likelihood ratio; TP = true-positive; FP = false-positive; FN = false-negative; TN = true-negative; ↑ indicates KCN for values higher or equal to the cutoff while ↓ indicates the presence of KCN when values are lower than the cutoff.

## Data Availability

The data presented in this study are part of an ongoing PhD study and are available on request from the corresponding author.

## Abbreviations

The following abbreviations are used in this manuscript:

K1a	front keratometry in flat meridian
K2a	front keratometry in steep meridian
Kmax	maximum keratometry front
K1pback	back keratometry in flat meridian
K2pback	back keratometry in steep meridian
Kmeanp back	mean keratometry posterior
VolC	corneal volume
TL	thinnest location
CCT	central corneal thickness
PI	pachymetric progression indices
ARTMax	maximum Ambrósio relational thickness
ISV	index of surface variance
IVA	index of vertical asymmetry
KI	KC index
CKI	central KC index
IHA	index of height asymmetry
IHD	index of height decentration
KISA%	keratoconus percentage index
ARC	anterior radius of curvature in the 3 mm zone
PRC	posterior radius of curvature in the 3 mm zone
I-S value	inferior-superior difference value
ARTh	Ambrósio’s relational thickness to the horizontal profile
DaRatio	deformation amplitude ratio 1 mm
BAD	Belin–Ambrósio ectasia display
BCVA	best corrected distance visual acuity
CBI	Corvis biomechanical index
BAD-D	Belin = Ambrósio enhanced ectasia total deviation value
TBI	tomographic and biomechanical index
A1L	length at the first applanation
A2L	length at the second applanation
A1Time	first applanation time
A2Time	second applanation time
A1Velocity	velocity at the first applanation
A2Velocity	velocity at the second applanation
SPA1	stiffness parameter at first applanation
DARatioMax1mm	the maximal value of the ratio between the deformation amplitude at the apex and at 1 mm from the corneal apex
DARatioMax2mm	the maximal value of the ratio between the deformation amplitude at the apex and at 2 mm from the corneal apex
HC PK	highest concavity peak distance
A1Dfl	the deflection length of the first applanation
A2Dfl	the deflection length of the second applanation
DefAmpMax	maximum deformation amplitude at the corneal apex
A1DeflectAmp	the deflection length of the first applanation
A2DeflectAmp	the deflection length of the second applanation
RMSTotal	root mean square total
RMS-HOA	root mean square high order aberration
Se	sensitivity
Sp	specificity
Acc	accuracy
+LR	positive likelihood ratio
-LR	negative likelihood ratio
TP	true positive
FP	false positive
FN	false negative
TN	true negative
AUC	area under the ROC curve
ROC	receiver operating characteristic
